# Association of Maternal Consumption of Ultra-Processed Foods with Feeding Practices and Malnutrition in Breastfed Infants: A Cross-Sectional Study

**DOI:** 10.3390/ijerph22040608

**Published:** 2025-04-12

**Authors:** Juliana Morais de Sousa, Danielle Soares Bezerra, Lara Virginia Pessoa de Lima, Priscila Gomes de Oliveira, Nicolie Mattenhauer de Oliveira, Elias Kelvin Severiano de Araújo, Lígia Rejane Siqueira Garcia, Juliana Fernandes dos Santos Dametto, Karla Danielly da Silva Ribeiro

**Affiliations:** 1 Post Graduate Program in Nutrition, Federal University of Rio Grande do Norte, Natal 59.078-970, RN, Brazil; juliana@nei.ufrn.br (J.M.d.S.); laravirginiapessoa@gmail.com (L.V.P.d.L.); pgomes.nutri@gmail.com (P.G.d.O.); elias.kelvin.109@gmail.com (E.K.S.d.A.); 2 Health Sciences College of Trairi, Federal University of Rio Grande do Norte, Santa Cruz 59200-000, RN, Brazil; danielle.bezerra@ufrn.br (D.S.B.); ligia.garcia@ufrn.br (L.R.S.G.); 3 Independent Researcher, Natal 59141-185, RN, Brazil; ni.mattenhauer@gmail.com; 4 Department of Nutrition, Federal University of Rio Grande do Norte, Senador Salgado Filho Avenue, University Campus, Natal 59.078-970, RN, Brazil; juliana.dametto@ufrn.br

**Keywords:** Nova classification, maternal nutrition, stunting, child nutrition, overweight, breastfeeding, lactation

## Abstract

Although the adverse health effects of consuming ultra-processed foods (UPFs) are well-documented, limited evidence exists on their impact during lactation. This study examined the association between maternal UPF consumption, feeding practices, and malnutrition in breastfed infants. A cross-sectional analysis was conducted with 111 mother–child pairs up to 150 days postpartum. Infant feeding practices were assessed using WHO indicators, and malnutrition was evaluated by length-for-age and BMI-for-age. Maternal dietary intake was estimated using two 24 h recalls, and UPF consumption was classified by the Nova classification. Dyads were grouped based on the highest UPF consumption quartile, and adjusted binary logistic regression was applied. UPFs accounted for 26% of the maternal diet on average. While 71.2% of infants were exclusively breastfed, one-third were overweight, and 11.7% were stunted. High maternal UPF consumption (>32% of energy) was associated with increased odds of malnutrition by BMI-for-age (wasting or overweight) (OR 3.38; 95% CI: 1.29–8.83) and stunting (OR 3.89; 95% CI: 1.04–14.58). Feeding practices showed no association. These findings highlight that maternal UPF consumption is associated with malnutrition odds in breastfed infants in the population assessed, emphasizing the need for dietary guidance during lactation to improve infant health outcomes.

## 1. Introduction

Ultra-processed foods (UPFs) account for over half of the dietary energy consumed in the United States, Canada, and the United Kingdom and one-third in Brazil [1,2,3]. High UPF consumption has been associated with poor diet quality, overweight, noncommunicable chronic diseases (NCDs), depression, and all-cause mortality across all life stages [4,5,6,7,8,9,10,11,12].

Despite growing interest in UPF consumption patterns, few studies have addressed its impact during lactation. A systematic review identified only one study evaluating UPF intake and health outcomes during this period. This Brazilian study found that 16% of lactating women’s energy intake came from UPFs, which impacted alpha-tocopherol levels and increased vitamin E inadequacy in breast milk, although infant outcomes were not assessed [6,13].

The first 1000 days of life are critical for growth and development, influenced by genetic, neuroendocrine, and environmental factors, such as nutrition and maternal bonding [14,15]. Diet during this period shapes long-term health, including risks for obesity, diabetes, and cardiovascular diseases through metabolic programming [16,17]. The early introduction of UPFs during the first year of life was associated with the double burden of malnutrition [18]. Maternal diet quality has been associated with infant feeding practices and growth outcomes [19,20,21].

Maternal UPF intake during pregnancy and early postpartum correlates with higher UPF consumption in children aged 6–24 months [21]. Breastfeeding reduces the likelihood of UPF inclusion in infant diets, reinforcing its role in modulating eating behaviors alongside its nutritional benefits [22]. However, these studies focus on children older than six months, leaving the relationship between maternal UPF consumption, breastfeeding practices, and malnutrition in younger infants unexplored.

Evidence also links maternal UPF consumption to adverse outcomes in pregnancy. An increase in UPF energy intake during pregnancy was associated with the occurrence of neonatal hypoglycemia [23] and higher neonatal adiposity [24], and similar trends were observed in lactating women [15]. Longitudinal studies in children reveal that high UPF intake predicts increased BMI trajectories and sustained adiposity into adulthood [25].

Given the adverse health effects of UPF consumption, the scarcity of research on its impact during lactation, and the importance of this period for maternal and child health, this study aimed to evaluate the association between maternal UPF consumption, infant feeding practices, and malnutrition in 0–6-month-old infants.

## 2. Materials and Methods

### 2.1. Study Design and Population and Ethical Aspects

This is a cross-sectional study developed with the mother–child dyads assisted in child growth and development care in the primary health care network in Natal, RN, Brazil. This study is part of a larger research project entitled “Influence of consumption of ultra-processed foods on the nutritional status and antioxidant profile of breastfeeding women and their infants in the city of Natal, RN” (Process No. 28/2018, CNPQ), which was submitted and approved by the Ethics and Research Committee of the Federal University of Rio Grande do Norte (UFRN) (CAAE 29928420.7.0000.5292), under code 4.199.673, and conforms Declaration of Helsinki. Before data collection, the research objectives and procedures were explained to the breastfeeding women in language accessible to their educational level. The document was then read in full to obtain the signature of two copies of the Informed Consent Form. One of the Informed Consent Forms was later given to each of the participants, and the other remained with the research team.

### 2.2. Sample Calculation

**Figure 1:**
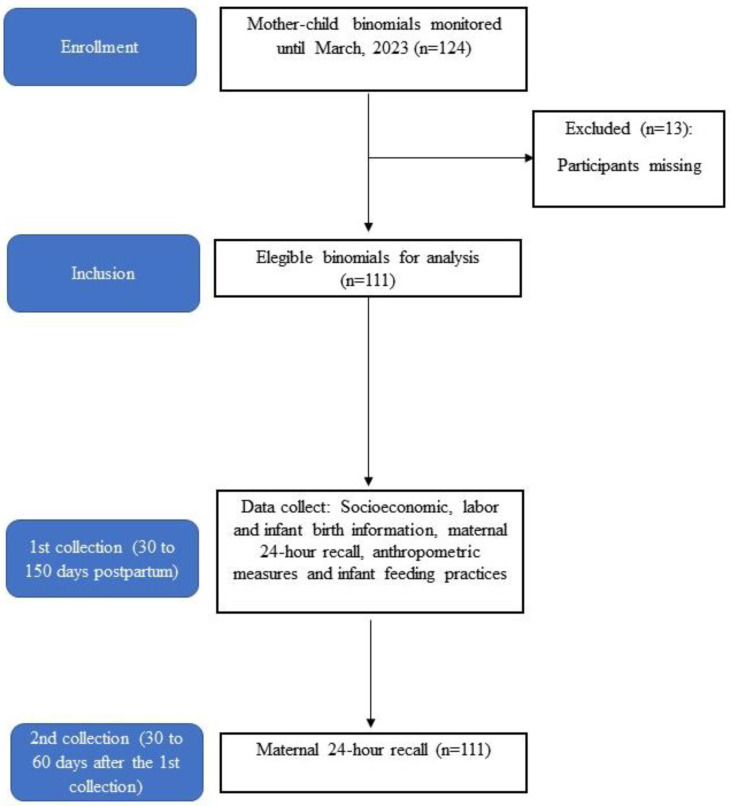
Flowchart of mother–child dyads through the study and data collection. Natal, RN, Brazil, 2021–2023 (*n* = 111).

The definition of the population was based on the sample analysis calculation for a difference between UPF consumption proportions in the breastfeeding women considered in the study conducted by Amorim et al. [13], with lactating women from the same city, adopted for the calculation of maternal UPF consumption of the lowest tertile (0–8.6%) and the highest tertile (>53.9%). A confidence interval of 95% was adopted, with 80% power, with a sample of 119 dyads being defined for this analysis (Figure 1) [26,27]. Due to the public health emergency of international concern resulting from the outbreak of the new coronavirus (COVID-19), data collection for the study was interrupted at times, and the access of the child population to health monitoring was also compromised. Therefore, all interviews carried out from July 2021 to March 2023 were considered, which resulted in 124 mother–child binomials. Of these, 111 were eligible for analysis. The posteriori sampling power of 61% was calculated considering exposed children (quartile 4%UPF) vs children quartile 1–3%UPF and the difference between the presence of any malnutritional according to BMI-for-age.

### 2.3. Recruitment, Eligibility Criteria, and Data Collection

All dyads that were present at the health center on the days of data collection were recruited to participate in the study, during their appointments for the Child Growth and Development Program or in a vaccination waiting room. We considered eligible the dyads consisting of adult women (>18 years old), who had full-term deliveries (≥37 gestational weeks) with a single conceptus and without malformation. Dyads that did not have data from 24 h dietary recall (24HR) or complete anthropometric data were excluded from the study.

### 2.4. Data Collection

The data collection occurred in nine primary health care centers in the four health districts of Natal, RN, from July 2021 to March 2023, totaling 124 mother–child dyads. After 30 to 60 days of the first data collection, a new one-on-one 24HR was administered to the lactating women at the health centers, at their residences, or by contact via telephone.

In the first stage of data collection, an interview was conducted with the breastfeeding women guided by a semi-structured electronic form, which included four dimensions: (a) socioeconomic data, (b) anthropometric data of the infant, which were measured by the researchers (weight and length), (c) infant dietary feeding, and (d) 24HR of the mother–child dyad. Weight was measured using a pediatric digital anthropometric scale (Welmy^®^, São Paulo, Brazil), and length was measured using a pediatric anthropometric ruler (Generalmed^®^, São Paulo, Brazil).

In the second stage of data collection, the anthropometric assessment of the infant was once again carried out, as well as the new collection of the dietary practices of the infant and new 24HR of the mother-child dyad.

### 2.5. Evaluation of Maternal Consumption of UPF

Food consumption data were collected using one adapted 24HR (RecNova24h) [28] applied at two points (no weekend) during the research: basal and after 30 to 60 days of the first data collection, using the multiple-pass method with the support of the Globo Diet photographic manual [29]. The instruments were administered by previously trained dietitians and nutrition undergraduate students. A pilot study was conducted to assess the instruments’ applicability.

The Virtual Nutri software (http://www.virtualnutriplus.com.br/Portal/Default.aspx accessed on 6 April 2025) was used to build a food database. The energy intake was analyzed based on the macronutrient information through the Brazilian Food Composition Table (TBCA), except when nutrient information was not available in that publication. In that case, the United States Department of Agriculture (USDA) food equivalents were used. Each food was categorized according to the four groups of the Nova classification, according to the purpose of processing. UPFs were categorized when on the verification of the labels of consumed food were observed isolated food parts containing flavorings, dyes, and emulsifiers or other food additives [30]. Then, the energy participation of UPFs in relation to the total dietary energy intake was obtained by two RecNova24h. The intra-individual variation was corrected by the multiple source method (MSM) (https://msm.dife.de/ accessed on 20 July 2023). Mother–child dyads were grouped according to the highest UPF consumption quartile (75th percentile), resulting in two groups: Q1–3 (first to third quartile) and Q4 (fourth quartile).

A ranking of the most consumed UPFs was evaluated by the sum of the calories accounted by each food and its participation in the total calories of the UPFs consumed by the studied population. For that, a cross-multiplication method (rule of three) was used. Thus, the UPFs were grouped by similarity, based on the study by Louzada et al. [4] with some adaptations. The foods were grouped according to shared characteristics, facilitating their classification. The meat products refer to the sausage, hot dogs, and mortadella groups; cookies/crackers include simple sweet cookies, stuffed cookies, savory cookies, and bakery cookies; bread refers to sliced bread, hamburger buns, and industrialized cheese bread.

### 2.6. Indicators of Infant Feeding Practices (Dependent Variables)

To evaluate the feeding practices of the infants, six breastfeeding indicators from the World Health Organization (WHO) [31] were used, as follows: the early initiation of breastfeeding (EIBF), exclusively breastfeeding for the first two days after birth (EBF2D), exclusively breastfeeding (EBF), mixed-milk feeding (MixMF), and bottle feeding (BoF). The information was confirmed by the 24HR administered to investigate infant feeding practices. The breastfeeding women answered yes or no to each listed indicator. EBF was considered when children consumed breast milk exclusively or with multivitamin/mineral supplements. Infants who consumed animal milk and/or milk formula, tea without milk, and/or water in addition to breast milk were categorized as MixMF. Breastfeeding practices were considered inadequate when there were negative responses to the EIBF, EBF2D, and EBF indices and positive responses to MixMF and BoF. Based on the presence of MixMF, another practice indicator was derived to investigate the duration of exclusive breastfeeding (DBF), which was considered short when the children were exclusively breastfed for less than 60 days, and long when the DBF was ≥days, based on the average number of days of exclusive breastfeeding for infants in the northeast of Brazil [32].

### 2.7. Evaluation of Malnutrition in Infants (Dependent Variables)

The infant’s weight and length measurements were taken according to the techniques proposed by Gilbride et al. [33] and a team of researchers trained in anthropometry. The children were weighed completely undressed, using a pediatric digital scale by Balmak. The length was obtained using an infantometer with the children in dorsal decubitus, with their head in the Frankfurt horizontal plane. Each anthropometric measurement was meticulously checked twice in each cycle; if a discrepancy greater than 1 cm was detected, the measurement was repeated once more. The length-for-age (LAZ), weight-for-age (WAZ), and BMI-for-age (BAZ) indices z-scores were calculated using the WHO Anthro software version 3.2.2 (https://www.who.int/tools/child-growth-standards/software, (Accessed on 20 May 2024) [34]. Malnutrition was considered in the following four situations: (a) when BAZ < −2 (wasting) only; (b) BAZ > +1 (overweight/obesity or risk for overweight) only, (c) some sort of malnutrition (wasting or overweight/obesity by BAZ); and (d) LAZ < −2 (stunting) only. WAZ was not used, because none of the children were underweight. The anthropometric classification employed in this study was guided by the protocol established by the Brazilian Ministry of Health, which delineates this cutoff point. The risk classification for overweight falls within the malnutrition category, as it signifies the necessity for nutritional intervention in cases where it manifests (http://189.28.128.100/nutricao/docs/geral/protocolo_sisvan.pdf, accessed on 20 August 2023).

### 2.8. Covariates

Information regarding maternal education and income, as well as the infant’s birth, was obtained through an interview with the breastfeeding mother and recorded in the electronic questionnaire. Maternal schooling level was categorized as low schooling when they had completed up to primary school, and as formal schooling when they had completed high school. Regarding the assessment of income, per capita family income was obtained by dividing the informed family income value by the number of residents in the household. The poverty line was considered when per capita income was less than half the general minimum wage (USD 112.97) in Brazil in the reference year (2021) [35].

### 2.9. Statistical Analysis

Data analysis was performed using the Statistical Package for the Social Sciences (IBM SPSS^®^) version 20.0 for Microsoft Windows^®^. The distribution normality of the variables was verified using the Kolmogorov–Smirnov test. A descriptive analysis of quantitative variables with normal distribution was presented as mean and standard deviation. Categorical variables were presented by absolute and relative frequencies.

For each lactating woman, the UPF energy percentage in the total diet was calculated, so that the dyads could be grouped and evaluated according to the quartiles of energy participation of UPFs in the maternal diet, using quartile 4 as a reference for the exposure variable. So, for statistical analyses, dyads were grouped into quartile 1–3 (Q1–3) and quartile 4 (Q4).

Descriptive statistics were used to determine the population profile according to maternal–infant demographic characteristics, infant feeding practices, infant anthropometric indices, and maternal food consumption. In addition, the socioeconomic status of the household was detailed at this stage of the analysis. The *t*-test was used for comparing the mean values of the continuous assessed differences in proportions for continuous variables and Pearson’s chi-square test assessed differences in proportions for categorical variables.

Subsequently, crude and adjusted binary logistic regression models were used to assess the association between Q4 (exposure variable) with inappropriate eating practices (yes or no) and the presence of malnutrition, considering the following negative outcomes: (a) some malnutritional according to BAZ (wasting or overweight/obesity), (b) the presence of wasting, (c) the presence of overweight/obesity; (d) the presence of stunting; and (e) the presence of inadequate feeding practices.

These analyses were adjusted for the following covariables: per capita income, maternal education, infant’s sex, the presence of EBF, weight at birth, and length at birth. Covariates were introduced into the models based on evidence of their potential role as confounders. The variables that were significant after the stepwise selection method remained in the final models, having an estimation of odds ratio and respective 95% confidence intervals.

The presence of outliers and analysis of randomness of missing values were analyzed using the Little’s Missing Completely at Random (MCAR) test, with imputed missing values (<1%) for performing the linear regression. Statistical significance was assessed through two-sided *p*-value < 0.05.

## 3. Results

**Figure 2:**
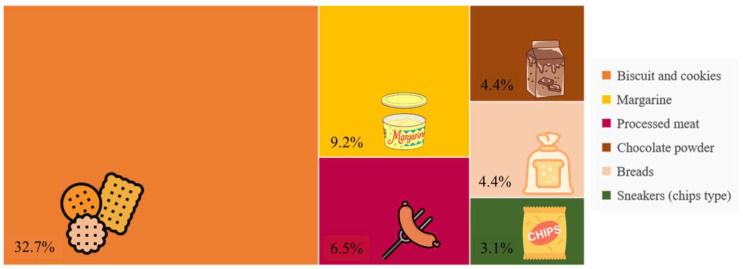
Main ultra-processed foods observed according to energy participation (%) in the diet of lactating women. Natal, RN, Brazil, 2021–2023 (*n* = 111).

The study population consisted of young women (mean age 28 years), predominantly from low-income households (66.7%), with over one-third having a low level of education (Table 1). UPFs accounted for 26% of the maternal diet (748.1 kcal, 95% CI: 0–1834.7), primarily from biscuits and cookies, margarine, and processed meats (Figure 2).

**Figure 3:**
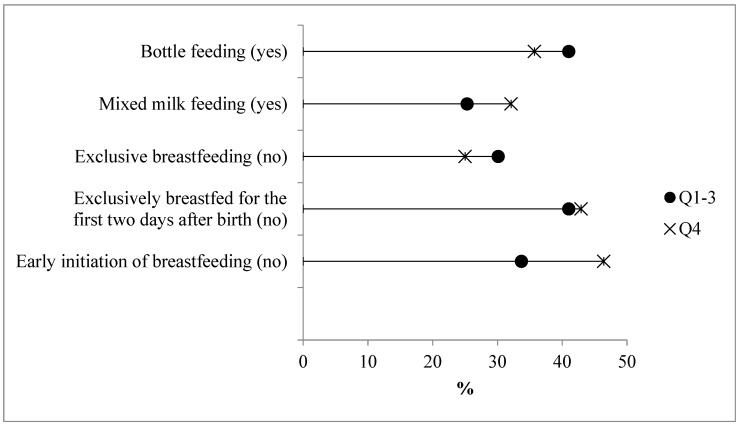
Proportion of infants with inadequate feeding practices according to participation of UPFs in maternal diet (Q1–3 vs Q4). Natal, RN, Brazil, 2021–2023 (n = 111). *p* > 0.05.

Infants had a mean age of 61 days, with 71.2% (*n* = 79) exclusively breastfed (EBF) and 28.8% (*n* = 32) receiving mixed feeding (MixMF). In the highest quartile of maternal UPF consumption, there was a higher proportion of infants with inadequate feeding practices, such as not initiating breastfeeding early (EIBF), shorter durations of exclusive breastfeeding (EBF2D), and MixMF use (Figure 3). However, no significant associations were found between high maternal UPF intake and inadequate feeding practices in infants (Table 2).

The prevalence of malnutrition (stunting, wasting, overweight/obesity, or malnutrition by BAZ) was higher among infants whose mothers were in the highest quartile of UPF consumption compared to lower quartiles (Table 3).

Binary logistic regression analysis (Table 4) revealed a significant association between the highest quartile of maternal UPF intake and an increased risk of wasting or overweight/obesity by BAZ (adjusted OR = 3.38; 95% CI: 1.29–8.83). Additionally, these infants had a nearly fourfold higher risk of stunting (low length-for-age; adjusted OR = 3.89; 95% CI: 1.04–14.58), adjusted for per capita income, maternal education, birth weight and length, and exclusive breastfeeding. A trend was observed towards a higher likelihood of overweight (BAZ) with greater maternal UPF consumption, though the difference was not statistically significant.

Infants born to mothers with high UPF consumption (Q4) were more likely to experience malnutrition as assessed by BAZ, particularly overweight/obesity, and had almost four times the risk of impaired growth (LAZ) compared to infants in the lowest maternal UPF consumption quartiles.

## 4. Discussion

Greater UPF maternal consumption was associated with stunting and wasting or excess weight among breastfed infants. We did not identify differences in infants’ feeding practices.

The caloric contribution of UPFs in the diet of lactating women (26%) exceeded the average observed in the Brazilian population (19.7%) [3] and the value reported in another study with Brazilian lactating women (16%) [13]. The pattern of UPF consumption was similar to that described by Amorim et al., where “breads, biscuits, and pasta” contributed 57% of the energy, followed by fats (13%) and processed meats (9%).

The temporal analyses of UPF consumption in Brazil indicate an increase among women, individuals with low education levels, and those with lower income, coupled with a decrease in the intake of unprocessed or minimally processed foods [36,37]. In this study, 57.1% of lactating women in the highest quartile of UPF consumption had low education levels, showing a significant association (*p* = 0.007) with the caloric contribution of UPFs in their diet.

Increased access to unhealthy foods among lower socioeconomic groups may be driven by lower prices and greater availability at purchase locations [38]. Peripheral neighborhoods, like those where most participants in this study lived, often face limited access to fresh and healthy foods, being categorized as food deserts (areas with scarce access to unprocessed or minimally processed foods) or food swamps (areas overexposed to establishments selling predominantly UPFs) [39]. Data collection for this study occurred during the COVID-19 pandemic, a period marked by behavioral changes, such as reduced physical activity, increased screen time, sedentary behavior, emotional eating, and the higher consumption of unhealthy foods. These factors were compounded by rising prices of unprocessed foods, poverty, and food insecurity [40,41,42]. Such circumstances likely contributed to the higher proportion of UPFs in the diets of the lactating women studied.

The results revealed that, in a low socioeconomic population, the infants of mothers with higher UPF consumption had increased odds of experiencing malnutrition, including overweight/obesity or stunting. However, no association was observed between maternal UPF consumption and indicators of infant feeding practices in infants under six months.

The literature on the role of maternal diet during lactation and its effects on infant growth and body composition is limited, complicating comparisons with our findings [6]. Amorim et al. [13] suggested a potential negative impact of high UPF consumption by lactating women on the nutritional quality of breast milk, emphasizing the importance of a balanced maternal diet for both the mother’s wellbeing and providing nutrient-rich breast milk to infants. Studies investigating UPF consumption during pregnancy or early childhood suggest associations with stunting (low LAZ), indicating that UPF intake may impair growth in young children. A cohort study in Ecuadorian children aged 6–9 months found that high UPF consumption tripled the risk of stunting (OR = 3), with similar results observed in Nepalese children aged 12–23 months [43,44].

Possible explanations for these associations include fetal overnutrition and the transgenerational cycle of obesity [45]. Maternal nutrition during prenatal and postnatal periods may induce epigenetic modifications affecting offspring metabolic function, growth hormone secretion, and appetite regulation [46,47], as well as altering the nutritional profile of breast milk [48].

The second hypothesis is that UPF composition is generally poor, containing sugars, refined starches, fats, and protein isolates, and contributes to an imbalanced diet characterized by excessive energy from carbohydrates, fats, and free sugars, along with deficiencies in vitamins, minerals, and fiber [1]. Therefore, the increased consumption of UPFs may result in a decreased diet quality and increased energy intake. Previous reports have associated deficiencies or excesses in a range of macro- and micronutrients with significant impairment in fetal development, excessive weight gain in pregnancy, and childhood obesity. UPFs are also a source of harmful compounds like endocrine disruptors (e.g., phthalates and bisphenols) [45,46,47,49]. The third hypothesis suggests that, beyond nutritional quality, UPFs have properties like additives and altered physical structure. The cumulative long-term effects of additives remain unclear [50]. Additives may impact gut microbiota composition and host–bacteria interactions, and food processing can change the food matrix, affecting nutrient absorption and microbiota growth [51]. Studies in rodents show that microbiota diversity loss from a Western diet can pass to future generations, highlighting the maternal diet’s role in offspring microbiota [52].

The coexistence of overweight/obesity (27.3%) and undernutrition (11.7%) in this study’s population highlights the double burden of malnutrition, typical of populations with low education levels, food insecurity, and socioeconomic disadvantages [53,54].

Although no significant correlations were found in this study, previous research demonstrates an association between high maternal UPF consumption and poor-quality dietary practices for children, such as the early introduction of unhealthy foods and reduced breastfeeding [21,55].

The mean duration of exclusive breastfeeding (EBF) for infants who had ceased EBF at the time of data collection was 51 days (SD 36.2), which is below the Brazilian Ministry of Health’s recommendation of 180 days and the median of 90 days observed in the Brazilian National Survey on Child Nutrition [56].

This study has some limitations inherent to the assessment of food consumption, such as fatigue during lactation, memory bias of the interviewee, and the food processing classification. However, some measures were taken to reduce these limitations, such as training the team for administering the 24HR and Nova classification; the use of a specific instrument for data collection on food consumption to obtain the food processing classification (RecNOVA24h); the use of the Globo Diet photographic manual to make the reported servings more reliable (closer to those actually consumed); and the breakdown of consumed dishes for a more careful assessment of ingested food and double checking the critical stages of consumption assessment. Also, the smaller number of people could be a limitation, which hindered the evaluation of the number of events in each group and added other variables in the logistic regression models, despite the sample power of 61%.

Among the positive traits of this study is its innovative theme on the impact of UPFs on health during a phase about which there are gaps in the literature—lactation. The observed associations highlight the importance of promoting the consumption of unprocessed or minimally processed foods, while discouraging UPF intake, in alignment with Brazilian and international dietary guidelines [57,58], in order to guarantee better health and prevent malnutrition in childhood and their negative long-term outcomes.

## 5. Conclusions

Maternal UPF consumption was associated with malnutrition in breastfed infants. Long-term studies are needed to further investigate the impact of UPFs on breast milk composition, maternal nutritional status, and subsequent childhood outcomes. Future research should focus on the first six months postpartum to provide new evidence on UPF consumption during lactation and its effects on child health.

## Figures and Tables

**Table 1:** Socioeconomic and maternal health characteristics and the birth profile of infants attended at Health Care Centers in Natal, RN, Brazil, according to quartiles of maternal consumption of UPFs.

Baseline Characteristics	Total (*n* = 111)	Ultra-Processed Food Consumption (% of Energy)		*p* Value
		Quartile 1–3 (*n* = 83)	Quartile 4 (*n* = 28)	
**Lactating women**				
Maternal age (years), mean (SD)	28 (6.7)	28 (6.7)	26 (6.7)	0.134
Postpartum days, mean (SD)	65 (35.7)	62 (34.8)	74 (37.8)	0.122
Pre-pregnancy BMI, mean (SD)	27 (6.0)	27 (6.4)	26 (4.9)	0.573
Current BMI, mean (SD)	28 (5.5)	28 (5.8)	27 (4.6)	0.712
Educational Level *				
Formal education, n (%)	71 (64%)	59 (71.1%)	12 (42.9%)	0.007 ^a^
Low education level, n (%)	40 (36%)	24 (28.9%)	16 (57.1%)	
Per capita income **				
No poverty, n (%)	37 (33.3%)	31(37.3%)	6 (21.4%)	0.122 ^a^
Poverty, n (%)	74 (66.7%)	52 (62.7%)	22 (78.6%)	
**Infants**				
Female, n (%)	48 (43.2%)	36 (43.4%)	12 (42.9%)	0.962 ^a^
Male, n (%)	63 (56.8%)	47 (56.6%)	16 (57.1%)	
Birth weight (g), mean (SD)	3353 (339.3)	3369 (383.5)	3307 (428.5)	0.477
Length at birth (cm), mean (SD)	49 (2.3)	49 (2.02)	48 (2.6)	0.100
DBF (days), mean (SD) ***	51 (36.0)	49 (36.3)	56 (35.0)	0.388 ^a^

* Education level: low educational level—up to elementary school; formal schooling—completed high school. ** Poverty line established based on the World Bank (½ minimum wage = USD 112.97). *** DBF—duration of exclusive breastfeeding, for those infants who were not on exclusive breastfeeding at the time of data collection. *t* test for independent samples; ^a^ Pearson’s chi-square test.

**Table 2:** Crude and adjusted logistic regression models for assessing the association between the highest quartile of participation of UPFs in the maternal diet and inadequate feeding practices of the infants attended at Health Care Centers in Natal, RN, Brazil (n = 111).

Feeding Practices of Breastfeeding	Consumption of Maternal UPFs (% of Energy—Quartile 4)			
	OR Unadjusted		OR Adjusted	
	OR (CI95%)	*p* Value	OR (CI95%)	*p* Value
EBF, no **	0.77 (0.29–2.05)	0.606	0.76 (0.27–2.14)	0.605
EIBF, no *	1.70 (0.71–4.07)	0.231	2.03 (0.79–5.23)	0.141
EBF2D, no *	1.08 (0.45–2.57)	0.860	1.05 (0.43–2.60)	0.913
MIXMF, yes *	1.40 (0.55–3.56)	0.482	1.47 (0.54–3.97)	0.448
BoF, yes *	0.80 (0.33–1.95)	0.801	0.86 (0.23–3.17)	0.815
DBF, no **	0.65 (0.25–1.74)	0.395	0.62 (0.21–1.81)	0.380

* Adjustment for covariates: per capita family income, education level, weight at birth and EBF; ** Adjustment for covariates: per capita family income, education level and birth weight.

**Table 3:** Assessment of malnutritional according to anthropometric indices in infants grouped by quartiles of maternal consumption of UPFs. Natal, RN, Brazil, 2021–2023 (n = 111).

Anthropometric Indicators	Total (n = 111)	Participation of UPFs in Maternal Diet (% of Energy)		*p* Value *
		Quartile 1–3 n = 83	Quartile 4 n = 28	
**Weight-for-age classification**				
Underweight for age, % (n)	1.8% (2)	2.4% (2)	0% (0)	0.497
Overweight for age, % (n)	1.8% (2)	2.4% (2)	0% (0)	
**Length-for-age classification**				
Stunting, % (n)	13 (11.7%)	7 (8.4%)	6 (21.4%)	0.064
**BMI-for-age classification**				
Wasting, % (n)	3 (2.7%)	1 (1.2%)	2 (7.1%)	0.037
Overweight/obesity, % (n)	33 (29.7%)	21 (25.3%)	12 (42.9%)	
**Presence of any malnutritional according to BMI-for-age**	36 (32.4%)	22 (26.5%)	14 (50%)	0.034

UPF—ultra-processed food; BMI—body mass index. * Pearson’s chi-square test. Malnutrition was evaluated by classifying BAZ > +1 (overweight) and BAZ < −2 (underweight).

**Table 4:** Logistic regression models for evaluating the association between the highest quartile of participation of ultra-processed foods in the mother’s diet and the presence of malnutrition in the infant. Natal, RN, Brazil, 2021–2023 (n = 111).

Malnutrition	Consumption of Maternal UPFs (% of Energy—Quartile 4)			
	OR Unadjusted		OR Adjusted	
	OR (CI95%)	*p* Value	OR (CI95%)	*p* Value
Wasting or overweight/obesity *	2.77 (1.14–6.73)	0.024	3.38 (1.29–8.83)	0.013
Wasting	6.31 (0.55–7.42)	0.139	10.81 (0.72–16.36)	0.086
Overweight/obesity	2.21 (0.90–5.43)	0.082	2.53 (0.97–6.61)	0.058
Stunting	2.96 (0.90–9.73)	0.074	3.89 (1.04–14.58)	0.044

* BAZ > +1 (overweight) or BAZ< −2 (wasting). OR = odds ratio. The adjustment variables were income per capita, mother’s education level, weight at birth, and EBF of infants. The variable “length at birth” was added in addition to the aforementioned to adjust “length at birth”.

## Data Availability

The data presented in this study are available on request from the corresponding author due to ethical reasons.

## Abbreviations

24HR	24 h dietary recall
BAZ	Body mass index for age z score
BMI	Body mass index
BoF	Bottle feeding
DBF	Duration of exclusive breastfeeding
EBF	Exclusively breastfeeding
EBF2D	Exclusively breastfeeding for the first two days after birth
EIBF	Early initiation of breastfeeding
LAZ	Length-for-age z score
MixMF	Mixed-milk feeding
OR	Odds ratio
Q1–3	First to third quartile
Q4	Fourth quartile
TBCA	Brazilian Food Composition Table
UFRN	Federal University of Rio Grande do Norte
UPFs	Ultra-processed foods
USDA	United States Department of Agriculture
WAZ	Weight-for-age z score
WHO	World Health Organization
